# Using Wind Tunnels to Predict Bird Mortality in Wind Farms: The Case of Griffon Vultures

**DOI:** 10.1371/journal.pone.0048092

**Published:** 2012-11-09

**Authors:** Manuela de Lucas, Miguel Ferrer, Guyonne F. E. Janss

**Affiliations:** 1 Department of Ethology and Biodiversity Conservation, Estación Biológica de Doñana (CSIC), Seville, Spain; 2 Fundación Migres, Seville, Spain; Plymouth University, United Kingdom

## Abstract

**Background:**

Wind farms have shown a spectacular growth during the last 15 years. Avian mortality through collision with moving rotor blades is well-known as one of the main adverse impacts of wind farms. In Spain, the griffon vulture incurs the highest mortality rates in wind farms.

**Methodology/Principal Findings:**

As far as we know, this study is the first attempt to predict flight trajectories of birds in order to foresee potentially dangerous areas for wind farm development. We analyse topography and wind flows in relation to flight paths of griffon vultures, using a scaled model of the wind farm area in an aerodynamic wind tunnel, and test the difference between the observed flight paths of griffon vultures and the predominant wind flows. Different wind currents for each wind direction in the aerodynamic model were observed. Simulations of wind flows in a wind tunnel were compared with observed flight paths of griffon vultures. No statistical differences were detected between the observed flight trajectories of griffon vultures and the wind passages observed in our wind tunnel model. A significant correlation was found between dead vultures predicted proportion of vultures crossing those cells according to the aerodynamic model.

**Conclusions:**

Griffon vulture flight routes matched the predominant wind flows in the area (i.e. they followed the routes where less flight effort was needed). We suggest using these kinds of simulations to predict flight paths over complex terrains can inform the location of wind turbines and thereby reduce soaring bird mortality.

## Introduction

An increase in the number of wind farms is currently in progress across the world [1]. Wind farms have received public and government support as alternative energy sources because they do not contribute to air pollution which is typically associated with fossil fuel technologies [2]. At the end of 2008, the global wind energy capacity surged by 28.8% and the total installed capacity reached 120.8 GW. Spain is the world's third largest wind energy market with 16.8 GW of installed electric generation capacity [3].

Nevertheless, like any other industrial activities entailing the use of land or sea, wind energy development inevitably has an ecological footprint that needs to be considered and addressed where relevant. Wind farms can affect birds mainly through fatal collisions with turbine blades [4]–[7] or through disturbance displacement [1], [8]. Although low collision rates have been recorded at many wind farms [9]–[11], some poorly-sited wind farms have had high collision mortality rates [11] and the potential for wind farms to cause problems for bird populations should not be underestimated [12], [13]. Currently, there is a high level of uncertainty when predicting the number of potential avian fatalities at proposed wind power developments [14].

There is a degree of consensus that raptors may be more vulnerable to collision than several other bird groups [13], [15], suggesting that their specific flight behaviour may contribute to turbine-related fatalities [4], [11], [16]. Visual field has been identified as another factor that could be involved in turbine collision [17], especially for *Gyps* vultures [18]. Of the raptor species inhabiting Spanish wind farm areas, the griffon vulture shows the highest mortality rates through collision [11], [16], [19] and de Lucas [11] recorded, between 1993 and 2003, 151 collisions in two wind farms located in Tarifa (southern Spain), 73% of which were griffon vultures.

Recently, Ferrer [20] showed that there are some weaknesses in the common methods used in risk assessment studies of proposed wind farms. Usually, these studies assume a linear relationship between the frequency of observed birds in the wind farm area and fatalities of birds [21]–[24]. Nevertheless, clear evidence exists showing that the probability of bird collisions with turbines depends critically on species behaviour and topographic factors, and not only on local abundance [11], [16], [20]. The main reason is that birds do not move over an area at random, but follow main wind currents which are affected by topography. Consequently, certain locations of wind turbines could be very dangerous even though there is a relatively low density of birds crossing the area whereas other locations could be very safe even with higher densities of birds [20]. This result challenges the main assumption of wind-farm assessment studies. If relevant factors affecting the frequency of collisions with turbine rotor blades are operating at the individual turbine scale, and not at the entire wind farm scale, environmental impact assessments must be focussed at the level of individual proposed turbines. In fact, variation in fatality rates among wind turbines within the same wind farm was more than double the variation between wind farms. Concentration of collision victims at few turbines in a wind farm, while nearby turbines that are superficially similar incur no deaths, indicates that “site selection” for turbines can play the most important role in limiting the number of collision fatalities [19]. Therefore a tool that detects the most dangerous locations for new wind turbines before the construction of the wind farm is urgently needed, in order to avoid these areas in future installations.

Flying birds moving over a landscape frequently exploit wind currents to assist their flight; this trait is particularly common in “soaring birds”, a diverse group, which includes several large raptors. Relief and related terrain features, change the horizontal and vertical air movements, which give important support to soaring flight movements [25]. Nevertheless, the influence of wind currents on local movements of large raptors has been rarely considered previously [26]. The overall premise is that soaring bird movements over a landscape are analogous to the distribution of wind currents. This is because soaring birds use pathways where the lowest effort is needed, taking advantage of thermals, ridge updrafts, and other sources of lift [27]. The griffon vulture is an archetypal soaring bird, and depends heavily on wind currents for major movements [25], [28], [29].

In this paper we test the null hypothesis that griffon vultures followed dominant wind currents by their local movements through a wind farm area in southern Spain to exploit the lowest energy cost flight path, and that these wind currents can therefore explain the distribution of vulture mortality between turbines. We used a three-dimensional model of a contour map of the wind farm area in a wind tunnel to determine where wind currents concentrated, and then compared these wind patterns with the flight routes used by griffon vultures according to field observations, and with the distribution of vulture mortality. The main aim of this study was to set the basis for future tools to predict potentially dangerous locations for wind turbines prior to the construction of wind farms.

## Materials and Methods

### Study area

PESUR wind farm is located in Tarifa, Andalusia region, south of Spain in the proximity of the Strait of Gibraltar. The Strait of Gibraltar is one of the most important locations for migrating Palearctic birds [30]–[32]. This area was the first region in Spain where turbines were installed, near Tarifa, and is one of four areas in Spain with the greatest potential for producing wind energy [33]. The vegetation in the study area was characterised by brushwood and scattered trees (*Quercus suber*, *Q. rotundifolia*) on the mountain ridges, with pasture land used for cattle grazing predominately in the lower areas.

**Figure 1:**
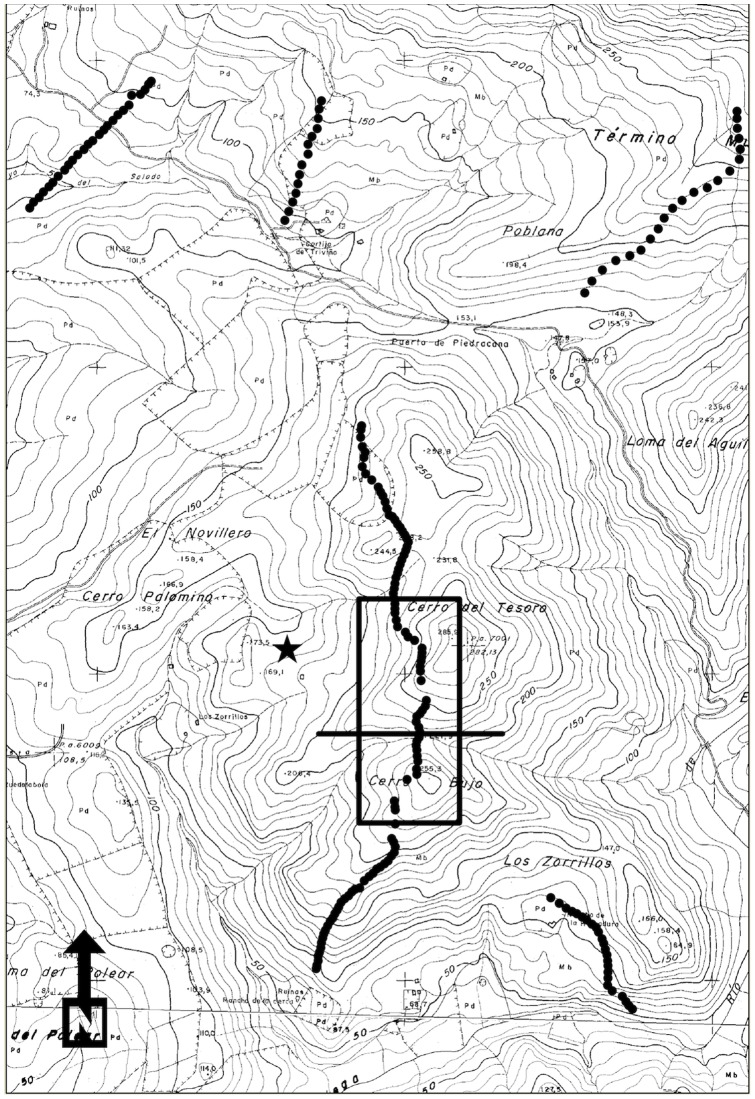
PESUR wind farm map (scale1∶10,000), illustrating the area covered by the scaled three-dimensional model and the observation point (indicated with a star). The valley is indicated by a straight line.

PESUR wind farm is situated in the Dehesa de los Zorrillos, on hills with a maximum elevation of 250 m a.s.l. (Figure 1). It contains 190 wind turbines in seven rows. We selected one of these rows for this study. Tesoro row is composed of 33 turbines, with two designs: AWP 56/100 (36 m tall lattice steel tower and 18 m diameter rotor) and AWP 56/100 (18 m tall lattice steel tower and 10 m diameter rotor). These AWP models made up a “wind wall” configuration consisting of wind turbines closely aligned to each other with alternating tower heights [4]. All rotors are orientated leeward and have three blades.

### Field methods

The study was carried out over four months (August, September, October and November) in 2002. Every second week of the month, observations were conducted on griffon vultures passing through the wind farm area, recording variables related to flight behaviour.

We selected a fixed observation point in the wind farm. A valley orientated from east to west, with two ridges orientated from north to south was selected. The Tesoro row of turbines was perpendicular to the valley (Figure 1) so that prevailing easterly and westerly winds optimised turbine operation.

Flight behaviour in the proximity of wind turbines (200 m height max. and 300 m width max.) was recorded by direct observation and by video cameras located at the fixed observation point. For each observation of an individual or group we recorded number of birds, climatic conditions (wind velocity and wind direction), flight direction and activity of the turbines. The flight trajectories were drawn on a map of our study area with an external 62.5×62.5 km grid. This permitted us to standardize and quantify the points where the vultures left our study area.

Mortality data were collected between November 1993 and June 2003 by staff of Department of Cadiz of the Andalusian Environmental Ministry. Each griffon vulture fatality record was associated with a carcass that was clearly attributable to a turbine collision rather than any other cause, and that did not share a body part with contemporaneous remains. From our previous experience we assumed that all dead birds the size of black kite or larger were found. The carcasses of such large birds were not lost to scavengers before searches, and were readily detected by human observers.

No specific permits were required for the described field studies, which did not involve endangered or protected species.

### Aerodynamic model

A very simple aerodynamic experiment was carried out in a low-speed open-circuit wind tunnel with a test chamber of 1.5 m width and 1.8 m height. This equipment is used in aerodynamic research to study the effects of air moving past solid objects (aircraft, buildings, vehicles, and birds [34]). A video camera located on the top of the wind tunnel was connected to a monitor to permit recording of the experiment. A wooden scaled model (1∶1,250) of the wind farm area was constructed with level curves each 12.5 m high (1 cm in the model) in the wind tunnel. Because air is transparent it is difficult to observe air movement directly. Hence, methods of flow visualization have been developed for testing in a wind tunnel. We used wool tufts attached and distributed regularly over the model to visualize surface wind flow and provide quantifiable data. A powerful upstream fan system moved air past the model and the pressure was equal to ambient at the exit. Tests were performed with several wind directions, including those which were most common (i.e., southerly, southeasterly, and easterly).

**Figure 2:**
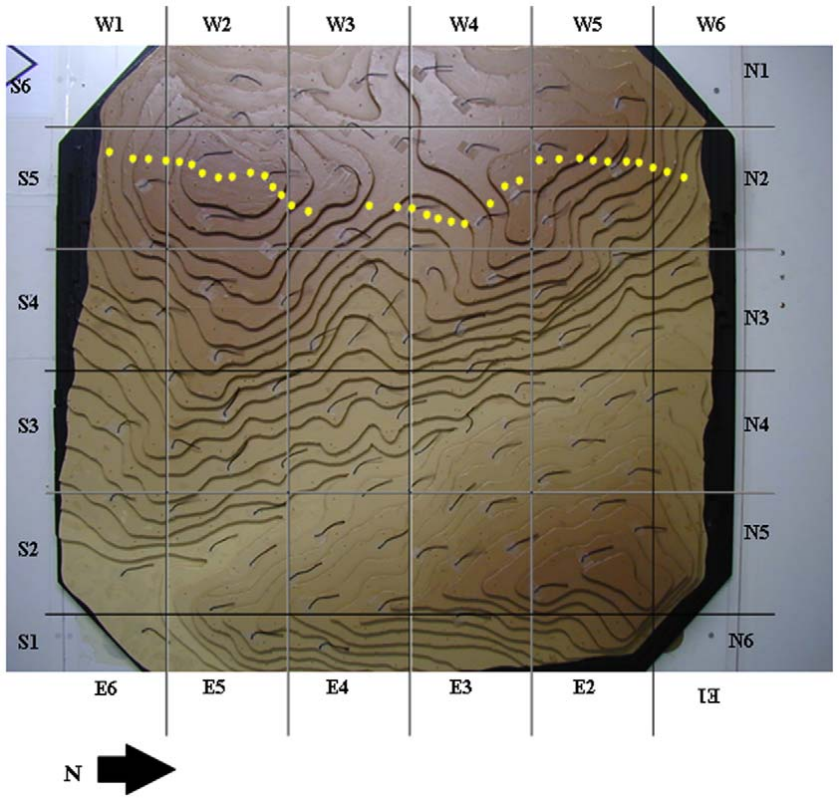
Scaled model (1∶1,250) with the wool plumes and showing the external grid. The study cells were used to define the points of departure of vultures and of wind currents from the study area. Yellow points indicate the study turbines.

The main wind passages (flows) in the scaled model were defined by observing the wool tufts. Arrows were drawn over the map to indicate the main streamlines (as one could expect, streamlines tend to concentrate at the saddles of the ridges, where the upstream air velocity increases). We added the same (scaled) external grid we used to define the flight routes of the vultures (5×5 cm; Figure 2) in the model and counted the number of arrow ends from the main wind passages in each cell to quantify the prevailing wind currents (none, one or two), such as where our model predicted the wind currents left our study area.

Due to the size of the grid in the aerodynamic model, we had to group several turbines inside the same grid cell. Mean number of turbines per cell was 5.5 and total number of cells with turbines was 6.

### Statistical methods

Chi-squared (goodness of fit) tests were used to compare the observed presence of vultures in each cell with: (1) an expected presence if flight routes were random (no preferred flight routes existed); and (2) an expected presence according to the moving wool plumes (flight routes coincided with wind passages). Spearman correlation was used to test relationship between accumulated mortality of the turbines included in each cell and the predicted proportion of vultures crossing each of these cells, based on the aerodynamic model. Statistica 6.0 software statistical package was used to perform statistical procedures and we used an alpha value of 0.05 to assess significance of results.

## Results

**Figure 3:**
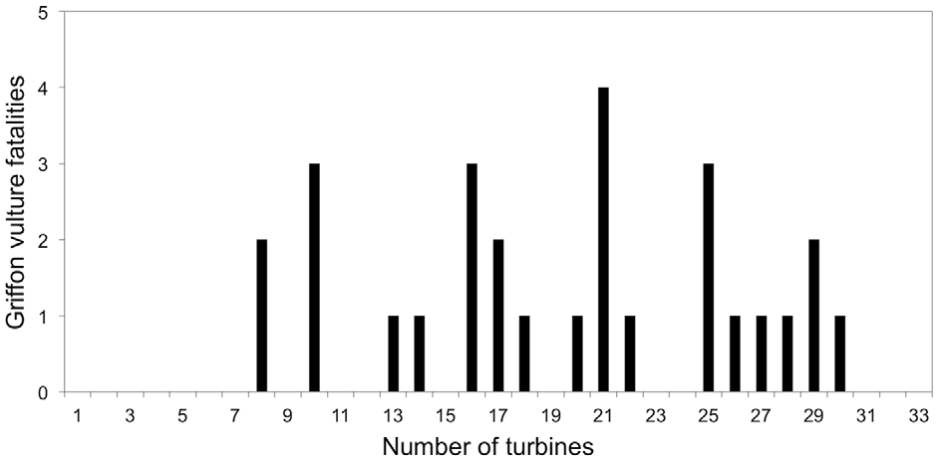
Distribution of the number of griffon vulture fatalities per turbine. The fatalities tend to be concentrated at certain turbines.

A total of 28 griffon vultures were found dead in the 33 turbines of the row (0.088/turbine/year). The distribution of griffon vulture mortality among the wind turbines was not uniform (Sign test, N = 33, Z = 5.570, p<0.001), showing a trend to be more concentrated at certain turbines (Figure 3).

**Figure 4:**
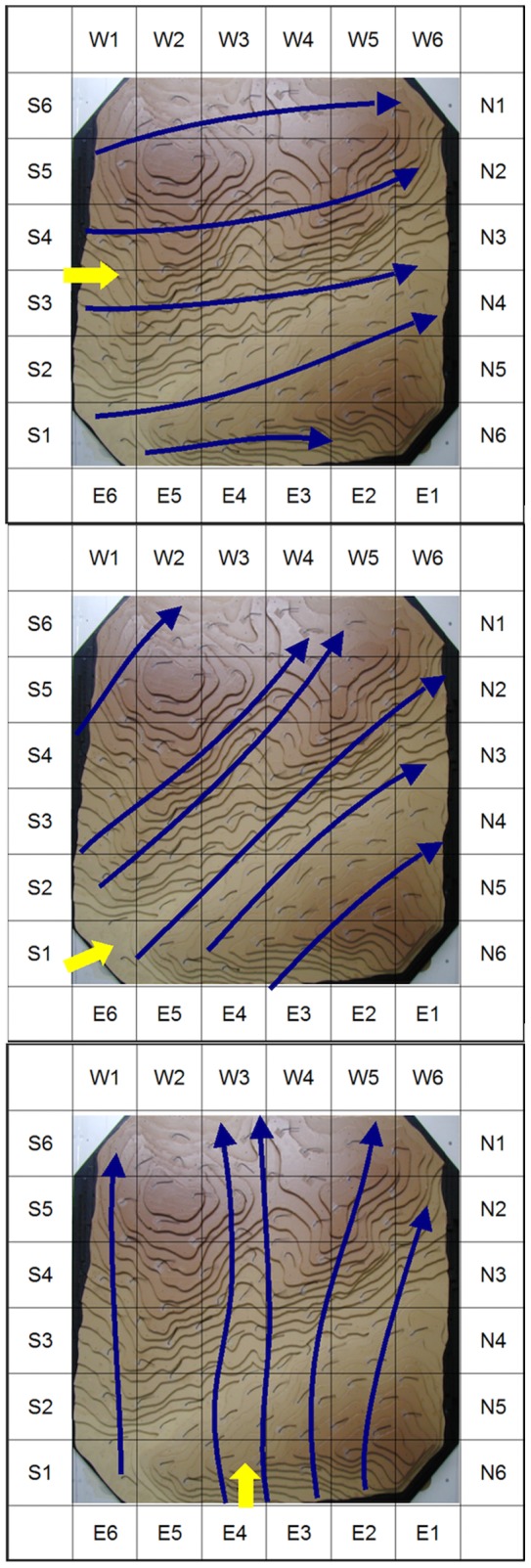
The three aerodynamic simulations conducted in the wind tunnel. The wind passages (flows) detected are indicated with blue lines. Yellow arrows indicate the simulated wind direction: A Southerly wind, B Southeasterly wind and C Easterly wind.

A total of 764 griffon vulture records were made during 176 hours of observation. 486 griffon vultures were observed on 10 days with easterly winds (Table 1), 63 griffon vultures were observed on four days with southerly winds and a 57 on four days with southeasterly winds. Different wind currents for each wind direction in the aerodynamic model were observed. Five wind passages with southerly winds, six wind passages with southeasterly winds and five wind passages with easterly winds were detected by studying wool plume movements (Figure 4).

**Table 1:** Total field observations.

	Eastern		Southeastern		Southern		TOTAL	
2002	N° days	N° birds	N° days	N° birds	N° days	N° birds	Days	Birds
August	3	206	1	11	2	25	6	242
September	2	28	2	29	1	14	6	74
October	2	59	1	17	1	24	5	110
November	3	193	0	0	0	0	5	338
TOTAL	10	486	4	57	4	63	22	764

With southerly winds, significant differences were detected between expected and observed flight directions (χ^2^ = 42.873, df = 9, p<0.0001), assuming a random vulture presence in all cells. When we used the flight directions from the wind tunnel model to calculate the expected values, no statistically significant differences were detected (χ^2^ = 4.682, df = 9, p = 0.861; Table 2). With southeasterly winds, significant differences were detected between expected and observed flight directions (χ^2^ = 79.466, df = 10, p<0.0001), assuming a random vulture presence in all cells. When we used the flight directions from the wind tunnel model to calculate the expected values, no statistically significant differences were detected (χ^2^ = 8.368, df = 10, p = 0.593; Table 3). With easterly winds, significant differences were detected between expected and observed flight directions (χ^2^ = 458.445, df = 9, p<0.0001), assuming a random presence in all cells. When we used the flight directions from the wind tunnel model to calculate the expected values, no statistically significant differences were detected (χ^2^ = 11.623, df = 9, p = 0.235; Table 4).

**Table 2:** Number of griffon vultures flying during field observations with southern winds and relative presence of wind currents observed in the aerodynamic model.

Cells	Field observation	Relative aerodynamic model
W4	0	0
W5	2	0
W6/N1	15	20
N2	7	20
N3	12	20
N4	8	20
N5	1	0
N6/E1	6	0
E2	0	0
E3	12	20
TOTAL	63	100

Locations where the vultures left the study area are indicated.

**Table 3:** Number of griffon vultures flying during field observations with southeaster winds and relative presence of wind currents observed in the aerodynamic model. We indicate the cells where vultures left the study area.

Cell	Field Observation	Relative aerodynamic model
S6/W1	4	0
W2	10	16,67
W3	0	0
W4	22	33,32
W5	0	0
W6/N1	6	0
N2	3	16,67
N3	5	16,67
N4	6	16,67
N5	0	0
N6/E1	1	0
TOTAL	57	100

**Table 4:** Number of griffon vultures flying during field observations with Eastern winds and relative presence of wind currents observed in the aerodynamic model.

Cell	Field observations	Relative aerodynamic model
S4	1	0
S5	5	0
S6/W1	1	0
W2	94	20
W3	120	20
W4	90	20
W5	95	20
W6/N1	0	0
N2	74	20
N3	6	0
TOTAL	486	100

We indicate the cells where vultures left the study area.

A significant correlation was found between dead vultures/number of turbines per cell and predicted proportion of vultures crossing those cells according to the aerodynamic model (r_s_ = 0.840, n = 6. P = 0.036), showing that higher mortality was recorded in those cells with higher expected proportion of vultures crossing.

## Discussion

Several studies of wind farm impacts on birds published in the scientific literature have focused on fatality rates [11], [16], [35]–[37]. However, to our knowledge, no data on flight trajectories have been published before, and as far as we know our study is the first to consider flight behaviour to predict areas of higher use by soaring birds.

Our results show that vultures were not moving at random over the area but following some trajectories more than others. These preferred trajectories were determined by the wind speed that was in turn related to the underlying topography. The observed flight trajectories of griffon vultures were not different to the wind passages predicted by our wind tunnel model (e.g. the vultures left our study area at the same points where highest wind velocities were reached), suggesting that griffon vultures use routes which are less energetically costly. Furthermore, a positive significant correlation was found between predicted proportion of vultures crossing cells with turbines and the vulture mortality records for these cells, showing that the distribution of these preferred wind currents was consistent with the distribution of vulture mortality between wind turbines.

Our study confirms and extends previous studies that have indicated a link between wind conditions, topography and flight behaviour as factors implicated in the spatial and temporal patterns of mortality of vultures within and between wind farms [11], [16], [19]. Soaring birds, such as vultures, do not move over a landscape at random, but follow the main wind currents, which are affected by topography at a small scale. The availability of wind currents enables cost-efficient flight in soaring birds, and so locations where wind flow is greatest are preferred, but these currents are also sought by wind energy development. Consequently, certain locations of wind turbines could be very dangerous even though there is a relatively low density of birds crossing the area whereas other locations could be very safe even with higher densities of birds in the wider area. If relevant factors affecting the frequency of collisions with turbine rotor blades are operating at the individual turbine scale, and not at the entire wind farm scale, environmental impact assessment must focus at the level of individual proposed turbines. In the future, it would be useful if such assessments would not only record the number of birds crossing proposed development sites but map bird flight paths at the scale of proposed individual turbines.

In addition to paying greater observational attention to a proposed turbine-level scale of bird flight activity, a potential new step in mitigation strategies to reduce bird mortality would be to conduct a test of a model of the proposed development area in a wind tunnel to determine, prior to construction, where the main concentrations of soaring birds are likely to occur. These models could be used to evaluate the relative effects of individual turbines within particular locations, using data from a meteorological mast recording wind speed and direction in the area. This kind of aerodynamic model, as well as any statistical model using existing wind and topographical data, if used at an early planning stage, could help to improve the process of selecting potential turbine locations and reduce the uncertainty over soaring bird mortality associated with wind farm development [20].

Overall the aerodynamic model results demonstrate that wind currents and three dimensional models are useful for simulating flight routes of soaring birds. The model yielded a valuable insight into observed flight patterns through a complex ridge-and-valley topographical system, and apparently helped explain why some turbines caused more fatalities than others. While our model was applied to a situation involving the local movements of griffon vultures, the principle has an obvious relevance to predicting sites of migratory raptor traffic.

## References

[1] Drewitt AL, Langston RHW (2006) Assessing the impacts of wind farms on birds. Ibis 148: 29–42.

[2] Huntley B, Collingham YC, Green RE, Hilton GM, Rahbek C, et al (2006) Potential impacts of climatic change upon geographical distributions of birds. Ibis 148: 8–28.

[3] Pullen A, Qiao L, Sawyer S (2008) Global Wind 2008 Report. Global Wind Energy Council. Brussels. 60 p.

[4] Orloff S, Flannery A (1992) Wind Turbine Effects on Avian Activity, Habitat Use, and Mortality in Altamont Pass and Solano County Wind Resource Areas. Sacramento: California Energy Commission. 145 p.

[5] Everaert J, Stienen EWM (2007) Impact of wind turbines on birds in Zeebrugge (Belgium). Biodivers. Conserv. 16: 3345–3359.

[6] Smallwood KS (2007) Estimating Wind Turbine-Caused Bird Mortality. J. Wildl. Manage. 71: 2781–2791.

[7] Thelander CG, Smallwood KS (2007) The Altamont Pass Wind Resource Area's effects on birds: a case history. In: de Lucas M, Janss G, Ferrer M, editors. Birds and Wind Farms. Madrid: Quercus Editorial. 25–46.

[8] Hötker H, Thomsen KM, Jeromin H (2006) Impacts on biodiversity of exploitation of renewable energy sources: the example of birds and bats – facts, gaps in knowledge, demands for further research, and ornithological guidelines for the development of renewable energy exploitation. Bergenhusen: Michael-Otto-institut im NABU. 65 p.

[9] Erickson WP, Johnson GD, Strickland MD, Young DP, Sernka KJ, et al.. (2001) Avian Collisions with Wind Turbines: A Summary of Existing Studies and Comparisons to Other Sources of Avian Collision Mortality in the United States. Washington: National Wind Coordinating Committee (NWCC). 62 p.

[10] Percival SM (2005) Birds and windfarms: what are the real issues? Br. Birds 98: 194–204.

[11] de Lucas M, Janss GFE, Whitfield P, Ferrer M (2008) Collision fatality of raptors in wind farms does not depend on raptor abundance. J Appl Ecol 45: 1695–1703.

[12] Hunt G (2002) Golden eagles in a perilous landscape: predicting the effects of mitigation for wind turbine blade-strike mortality. Santa Cruz: California Energy Commission. 72 p.

[13] Madders M, Whitfield DP (2006) Upland raptors and the assessment of wind farm impacts. Ibis 148: 43–56.

[14] Mabee TJ, Cooper BA, Plissner JH, Young DP (2006) Nocturnal Bird Migration Over an Appalachian Ridge at a Proposed Wind Power Project. Wildl. Soc. Bull. 34: 682–690.

[15] Hoover SL, Morrison ML (2005) Behaviour of Red-Tailed Hawks in a wind turbine development. J Wildl Manage 69: 150–159.

[16] Barrios L, Rodriguez A (2004) Behavioral and environmental correlates of soaring-bird mortality at on-shore wind turbines. J Appl Ecol 41: 72–81.

[17] Martin G (2011) Understanding bird collisions with man-made objects: a sensory ecology approach. Ibis 153: 239–254.

[18] Martin GR, Portugal SJ, Murn CP (2012) Visual fields, foraging and collision vulnerability in *Gyps* vultures. Ibis 154: 626–631.

[19] de Lucas M, Ferrer M, Bechard MJ, Muñoz AR (2012) Griffon vulture mortality at wind farms in southern Spain: Distribution of fatalities and active mitigation measures. Biol Conserv 147: 184–189.

[20] Ferrer M, de Lucas M, Janss GFE, Casado E, Muñoz AR, et al (2012) Weak relationship between risk assessment studies and recorded mortality in wind farms. J Appl Ecol 49: 38–46.

[21] Langston RHW, Pullan JD (2003) Wind farms and birds: an analysis of the effects of wind farms on birds, and guidance on environmental assessment criteria and site selection issues. Strasbourg: RSPB/BirdLife. 58 p.

[22] Smallwood KS, Thelander CG (2004) Developing methods to reduce bird mortality in the Altamont Pass Wind Resource Area. California Energy Commission, California. 520 p.

[23] Tapia L, Domínguez J, Rodríguez L (2009) Using probability of occurrence to assess potential interaction between wind farm and a residual population of golden eagle *Aquila chrysaetos* in NW Spain. Biodivers Conserv 18: 2033–2041.

[24] Tellería JL (2009) Wind power plants and the conservation of birds and bats in Spain: a geographical assessment. Biodivers Conserv 18: 1781–1791.

[25] Pennycuick CJ (1972) Soaring behaviour and performance of some east African birds, observed from a motor-glider. Ibis 114: 178–218.

[26] McLeod DRA, Whitfield DP, McGrady MJ (2002) Improving Predicting of Golden Eagle (*Aquila chrysaetos*) Ranging in Western Scotland Using GIS and Terrain Modeling. J. Raptor Res 36: 70–77.

[27] Pennycuick CJ (1998) Towards an optimal strategy for bird flight research. J. Avian Biol. 29: 449–457.

[28] Bildstein K, Bechard MJ, Farmer C, Newcomb L (2009) Narrow sea crossings present major obstacles to migrating Griffon Vultures *Gyps fulvus* . Ibis 151: 382–391.

[29] Bildstein K, Bechard MJ, Farmer C, Newcomb L. (In press) Flight behaviour of griffon vultures *Gyps fulvus* at a migration bottleneck. Ibis.

[30] Bernis F (1980) La migración de las aves en el Estrecho de Gibraltar. Madrid: Universidad Complutense de Madrid. 481 p.

[31] Finlayson C (1992) Birds of the Strait of Gibraltar. London: T. & A. D. Poyser. 420 p.

[32] Bildstein K, Zalles JL (2000) Raptor Watch: A Global Directory of Raptor Migration Sites. London: Birdlife Conservation. 320 p.

[33] IDAE (1996) Manuales de energías renovables 2. Energía eólica. Madrid: Cinco Días Editorial. 151 p.

[34] Pennycuick CJ, Alestarm T, Hedenström A (1997) A new low-turbulence wind tunnel for bird flight experiments at Lund University, Sweden. J. Exp. Biol. 200: 1441–1449. 10.1242/jeb.200.10.1441 9319339

[35] Musters CJM, Noordervliet MAW, Terkeus WJ (1996) Bird casualties caused by a wind energy project in an estuary. Bird Study 43: 124–126.

[36] Osborn RG, Higgins KF, Usgaard RE, Dieter CD, Neiger RD (2000) Bird Mortality Associated with Wind Turbines at the Buffalo Ridge Wind Resource Area, Minnesota. Am. Midl. Nat. 143: 41–52.

[37] Johnson GD, Erickson WP, Strickland MD, Shepherd MF, Shepherd DA, et al (2002) Collision mortality of local and migrant birds at a large-scale wind-power development on Buffalo Ridge, Minnesota. Wildl. Soc. Bull. 30: 879–887.

